# Process evaluation for the Care Homes Independent Pharmacist Prescriber Study (CHIPPS)

**DOI:** 10.1186/s12913-021-07062-3

**Published:** 2021-10-02

**Authors:** Linda Birt, Lindsay Dalgarno, David J Wright, Mohammed Alharthi, Jackie Inch, Maureen Spargo, Jeanette Blacklock, Fiona Poland, Richard C Holland, David P. Alldred, Carmel M. Hughes, Christine M. Bond, on behalf of the CHIPPS study team, Annie Blyth, Laura Watts, Amrit Daffu-O’Reilly

**Affiliations:** 1 The Queen’s Building, School of Health Sciences, University of East Anglia, Norwich Research Park, Norwich, NR4 7TJ UK; 2 Institute of Applied Health Sciences, University of Aberdeen, Aberdeen, UK; 3 School of Pharmacy, University of East Anglia, Norwich, UK; 4 School of Pharmacy, Queen’s University Belfast, Belfast, UK; 5 Leicester Medical School, University of Leicester, Leicester, UK; 6 School of Healthcare, University of Leeds, Leeds, UK; 7 NIHR Yorkshire and Humber Patient Safety Translational Research Centre, Leeds, UK

**Keywords:** Deprescribing, Care homes, Pharmacist, Implementation, Primary care, Older people medication

## Abstract

**Background:**

Medicines management in care homes requires significant improvement. CHIPPS was a cluster randomised controlled trial to determine the effectiveness of integrating pharmacist independent prescribers into care homes to assume central responsibility for medicines management. This paper reports the parallel mixed-methods process evaluation.

**Method:**

Intervention arm consisted of 25 triads: Care homes (staff and up to 24 residents), General Practitioner (GP) and Pharmacist Independent Prescriber (PIP). Data sources were pharmaceutical care plans (PCPs), pharmacist activity logs, online questionnaires and semi-structured interviews. Quantitative data were analysed descriptively. Qualitative data were analysed thematically. Results were mapped to the process evaluation objectives following the Medical Research Council framework.

**Results:**

PCPs and activity logs were available from 22 PIPs. Questionnaires were returned by 16 PIPs, eight GPs, and two care home managers. Interviews were completed with 14 PIPs, eight GPs, nine care home managers, six care home staff, and one resident. All stakeholders reported some benefits from PIPs having responsibility for medicine management and identified no safety concerns. PIPs reported an increase in their knowledge and identified the value of having time to engage with care home staff and residents during reviews. The research paperwork was identified as least useful by many PIPs. PIPs conducted medication reviews on residents, recording 566 clinical interventions, many involving deprescribing; 93.8% of changes were sustained at 6 months. For 284 (50.2%) residents a medicine was stopped, and for a quarter of residents, changes involved a medicine linked to increased falls risk. Qualitative data indicated participants noted increased medication safety and improved resident quality of life. Contextual barriers to implementation were apparent in the few triads where PIP was not known previously to the GP and care home before the trial. In three triads, PIPs did not deliver the intervention.

**Conclusions:**

The intervention was generally implemented as intended, and well-received by most stakeholders. Whilst there was widespread deprescribing, contextual factors effected opportunity for PIP engagement in care homes. Implementation was most effective when communication pathways between PIP and GP had been previously well-established.

**Trial registration:**

The definitive RCT was registered with the ISRCTN registry (registration number ISRCTN 17847169).

**Supplementary Information:**

The online version contains supplementary material available at 10.1186/s12913-021-07062-3.

## Background

Medicines management in care homes within the United Kingdom (UK) has been found to require significant improvement both from the perspective of quality assurance processes [1] clinical safety and effectiveness. The 2009 CHUMS study, in which medicines systems and their management process were observed in a large number of homes across England, identified that 70% of residents experienced at least one medication error every day, ranging from sub-optimal prescribing to inadequate medication monitoring, administration, storage and recording [2]. Problems with over-prescription of antipsychotics [3], laxatives [4], under-prescribing of analgesics [5] and unnecessary prescribing of medicines increasing the risk of falls [6] or anticholinergic burden [7] are all commonly reported within the international literature. Effective, safe and cost-effective models for enhancing pharmaceutical care and clinical outcomes within care homes have been identified as still requiring development [8, 9].

We proposed that the integration of a pharmacist with independent prescribing rights (pharmacist independent prescriber-PIP) into care homes to assume central responsibility for medicines-related activities might provide a model of care which would address both quality assurance and clinical concerns. The Care Home Independent Prescribing Pharmacist Study (CHIPPS) [10] was a programme of work which, in line with established guidance [11], developed and evaluated a complex pharmacist-led intervention of such a service. First, we developed a service specification through extensive stakeholder engagement [12]. Stakeholders agreed that the components of the PIP service should include medicine review (including deprescribing and minimising the potential for adverse effects from medicines) and pharmaceutical care planning, authorisation of monthly repeat prescriptions, supporting the optimisation of ordering and storage systems, supporting care home staff with medicines administration and training care home staff [13]. The PIPs were expected to become a central resource for all care home medicines activities and consequently, they required effective processes for communicating with the medical practice, care home and the supplying community pharmacy. The final service specification (additional file 1) included both ‘essential’ and ‘discretionary’ activities depending on identified local needs.

A training programme was developed to equip pharmacists with requisite knowledge and skills to assume responsibility for prescribing for a frail, older population, with a focus on reducing risk of falls. It comprised two taught days followed by 6 days of self-directed activities focussing on integration of PIPs into teams and collation of evidence for formal competency assessment and sign-off [14].

A logic model was developed in the initial stages of the project to propose a theoretical basis for the intervention. A standard approach was used to identify appropriate outcomes for the main trial. The final outcome set included medication appropriateness, adverse drug events, prescribing errors, falls, quality of life, all-cause mortality and admissions to hospital and a health economic assessment [15].

The service model and research design were subsequently tested in a feasibility study in four locations, each with one PIP, medical practice and cohort of ten care home residents (triad) [16]. Learning from this study resulted in a refined protocol for the definitive cluster randomised controlled trial comparing the effectiveness and cost effectiveness of a pharmacist independent prescriber managing care home residents’ medicines compared to usual GP-led care, with reduction in falls a primary outcome. The definitive trial was conducted across four sites within three of the four UK devolved nations, over 6 months. In the intervention arm, each PIP was allocated approximately 20 residents and reimbursed for the equivalent of four hours a week. To enable management of recruitment, training and data collection, the study was delivered over four phases within a two-year study period (2018–20).

To understand the exact way the intervention was implemented in practice, and the implications of this for the trial outcomes we designed a mixed-method process evaluation [17], following the Medical Research Council’s process evaluation framework [11]. The objectives were:

1. To describe the intervention as delivered in terms of quality, quantity, adaptations and variations across triads and time.
2. To explore the effects of individual intervention components on the primary outcomes.
3. To investigate the mechanisms of impact.
4. To describe the perceived effectiveness of relevant intervention components (including PIP training and care home staff training) from participant (GP, care home, PIP and resident/relative) perspectives.
5. To describe the characteristics of GP, care home, PIP and resident participants to assess reach.
6. To estimate the extent to which intervention delivery was normalised among the intervention healthcare professionals and related practice staff [17].

Quantitative trial data collected to describe the effectiveness of the intervention (falls, EQ-5D-5L, Barthel scores, drug burden index (DBI), mortality, hospitalisation and adverse events) (objective 2) are not reported here, but will be reported in the main RCT paper (submitted for separately). The process evaluation was undertaken prior to the analyses of the trial outcomes to ensure the researchers’ interpretations were not influenced by the results of the trial.

## Method

To address the evaluation objectives, this mixed-methods process evaluation used qualitative and quantitative approaches to describe the processes of intervention implementation, the mechanisms of impact, the outcomes and the contextual factors, in the intervention triads.

### Data collection, management, and analysis

We collated data on PIP, GP, and care home demographics across the four locations. Data analysis was an iterative process with each data set first analysed separately by specialist qualitative researchers before synthesis. The process evaluation objectives [17] were our anchor questions [18] guiding the analysis. Trial records and questionnaire results provided quantifiable data on PIP activity; we aligned this with the qualitative accounts to provide contextual background to numerical data on the facilitators and barriers to implementation, the mechanisms of carrying out the intervention, and perceived usefulness of the intervention outcomes.

#### Pharmaceutical care plans (PCPs)

During the intervention PIPs completed and returned pharmaceutical care plans which detailed biomedical monitoring requests, medicine review and actions at resident level. Three hundred and seventy PCPs are included in this process evaluation (see below for explanation of missing PCPs). Trial geriatricians reviewed a random 20% sample of PCPs, weighted to the start of each intervention period, to assess safety. Two research pharmacists within the research team reviewed 185 (50%) PCPs (105 reviewed independently in duplicate) for ‘potential missed opportunities’ based on the guidelines on appropriate prescribing used by the PIPs, which included checking that an appropriate indication, recommended biomedical monitoring and regular review of ongoing need had been recorded. The term ‘potential missed opportunity’ is used because without access to full resident notes and with no insight into conversations which might have taken place, there could have been valid reasons why a guideline recommended change had not been made. To explore the effects of individual intervention components on the primary outcomes, a doctoral student with a pharmacy qualification categorised the medication changes by British National Formulary (BNF) therapeutic group [19], by causal link to falls with Odds Ratios (OR) 1–1.5; 1.51–2; > 2 [20–22], and by Drug Burden Index (DBI) [23]. The DBI is (a dose-related measure of individual burden from anticholinergic and sedative drug exposure) (≤ 0.5; 0.51–1; 1.01–1.5). All were reported as frequencies.

#### Activity logs

The PIPs logged their use of time, categorised by activity type, on standard forms. The 10 categories were amalgamated into four groups: face-to-face time with resident; desk time on resident-related activity; other trial activity; and travel time. All were summarised using descriptive statistics.

#### Questionnaires

Post-intervention, an online questionnaire was sent via the REDCap data management system [24] to 88 stakeholders: 25 PIPS, 25 GPs, 38 care home managers. Likert scale measures and open text boxes were used to examine perceptions of training, variety and frequency of tasks delivered, opinions on usefulness of different parts of the service, team communication and relationships. The questionnaire also included the NoMad survey instrument [25] which captures data relevant to the four Normalization Process Theory domains [26]; NoMAD data is not reported here. The questionnaire was piloted on stakeholders from the feasibility study; no changes were made. The Likert scale responses were treated as ordinal data, with frequency across stakeholder groups displayed as bar charts. The open-text responses were added to the interview data on NVivo [27].

#### Interviews

Emails inviting all 88 stakeholders to interview were sent to the intervention triads: 25 PIPs, 25 GPs, 38 Care home managers (2 care homes recruited to the intervention were no longer contactable due to closure). Care home managers were asked to share the invitation to take part in an interview with care staff, residents and their relatives involved in the intervention. Following consent from each participant, semi-structured interviews were conducted which explored participants’ views of the PIP service (including barriers and facilitators to implementation, impact on workload, and working relationships focusing on communication, and acceptability). Topic guides (Additional file 2) were developed specific to each stakeholder group with those for residents and family reviewed by PPI members for clarity.

LD and LB undertook interviews either face-to-face or by telephone, between May 2019 and March 2020. Interviews were audio-recorded and transcribed verbatim. Data were thematically analysed drawing on the stepped approach suggested by Braun and Clarke [28]. Following familiarisation with the data, a coding framework was developed which aligned with the process evaluation framework and drew on principles of Normalization Process Theory. The coding framework was agreed within the multidisciplinary team. Coding was undertaken independently by LB and LD who met fortnightly to discuss differences in coding and come to a consensus. During, this process some codes were merged and others subdivided to explore similarities and differences across stakeholders.

At all stages of the analysis process including developing the coding framework, considering contextual differences in experiences, and searching negative cases the wider multidisciplinary study team, including clinical and academic pharmacists and GPs, were consulted.

## Results

Twenty-five intervention triads including 449 residents were recruited to the trial from sites in Scotland (9 triads), Northern Ireland (5) and England: Yorkshire (6), East Anglia (5). Three PIPs did not return any trial data and were recorded as not delivering the intervention; they were invited to complete the online questionnaire and an interview, but none responded. Of the 22 PIPs who provided the intervention for 6 months, 18 (82%) were female. The median time practising as a prescriber was 3 and half years with a range of 2 months to 16 years. Fifteen PIPs (68%) had worked in their triad GP practice prior to the trial. Twelve PIPs delivered the intervention in one care home, six PIPs across two care homes and four PIPs across three care homes; three of these were in Northern Ireland where care homes are often supported by several GP practices. Thirteen PIPs were allocated between 20 and 24 residents, 5 had 11–19 residents, 4 had 10 or less residents. Table 1 presents demographic data on sample. Additional file 3 contains full details of PIP, and GP practice and care home characteristics.

**Table 1:** Demographics by stakeholder

Recruited to:	Intervention	Post intervention questionnaire	Post intervention interview
**PIP** (Pharmacist independent Prescriber)			
Number of PIPs	25	16	14
Mean time as registered pharmacist	19 years (8–40 years)	21 years (8–40 years)	20 years (10–36 years)
Mean time as independent prescriber	56 months (1–192 months)	65 months (1–192 months)	58 months (1–192 months)
Mean number of residents allocated to the PIP	18 SD 5.96	18 SD 6.34	18 SD 6.34
Had previous care home experience	12 (48%)	6 (37.5%)	7 (50%)
**GP practices**			
Number of GP practices	25	8	8
Employed intervention pharmacist prior to study	15 (60%)	3 (37.5%)	4 (50%)
Registered patients ≥10,000	9 (36%)	3 (37.5%)	3 (37.5%)
**Care homes**			
Number of Care homes	40	2 care homes (2 staff)	11 care homes (15 staff)
Type of Care home	Residential 15 (37.5%) Dual 25 (62.5%)	Residential 1 (50%) Dual 1 (50%)	Residential 4 (36.3%) Dual 7 (63.6%)
Type of funding	Private 34 (85%) Local Authority 3 (7.5%) Other 3 (7.5%)	Private 1 (50%) Local Authority Other 1 (50%)	Private 9 (81.8%) Local Authority Other 2 (18.2%)
Mean number of residents in care homes	49 SD 18.83	40 SD 6.36	43 SD 13.09

Main trial records (PCPs and activity logs) were collected from 22 PIPs. Questionnaires were completed by 26 participants (30% return) across 21 intervention triads (PIP = 16; GP = 8; care home manager [CHM] =2). Interview data were collected from 38 participants across 18 triads (PIP = 14; GP = 8; CHM = 9; care home staff [CHS] = 6; Resident = 1). Eighteen participants completed both survey and interview (PIP = 13; GP = 3; CHM = 2). Interviews lasted between 20 and 70 min. In two triads, PIPs completed the intervention, but we were unable to collect questionnaire or interview data from any of the stakeholders. In the three triads where the PIP did not complete the intervention the only data collected was from one GP questionnaire.

Results are presented under four strands related to the process evaluation objectives: 1) Intervention implementation (including adequacy of PIP training and the fidelity of service delivery); 2) Mechanism of impact (description of individual intervention components which could have affected the trial outcomes); 3) Outcomes (PIP, GP and care home staff perception of impact of the intervention); 4) Contextual factors (descriptions of barriers and facilitators to delivering the intervention and adaptations and variations across triads). Illustrative quotes are drawn from open text boxes in the questionnaire and interview.

### Strand 1 intervention implementation (objectives 1,2,4)

#### Training for PIPs

In the questionnaire responses, 13 of 16 PIPs reported that the training was sufficient. Three PIPs reported it could be further enhanced by including more information on how to use the PCPs and one requested more examples of deprescribing. Having input from geriatricians and the opportunities to discuss case studies were reported as beneficial both in refreshing knowledge and in adding to their understanding of geriatric medication.

During interviews, all PIPs (*n* = 14) reported that the training, professional development and assessment against a competency framework led to them feeling confident and competent in their intervention role.

#### PIP delivery of intervention

##### Services provided

**Fig. 1:**
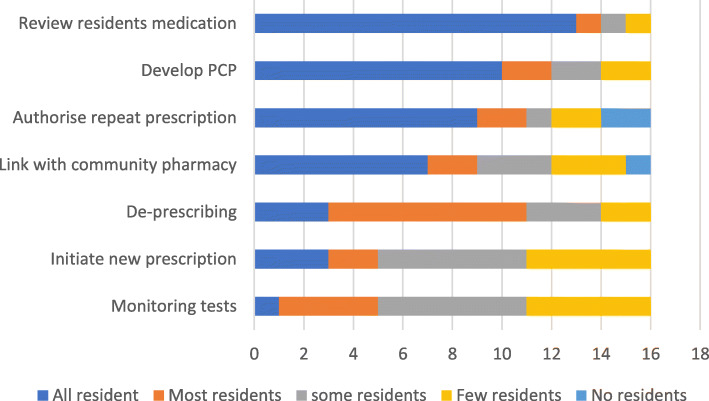
PIP (*n* = 16) reported delivery of essential activities

Questionnaire data indicated the extent that PIPs delivered each of the essential intervention activities. Medication review, pharmaceutical care planning and repeat prescription authorisation were frequently reported, with greater variation in the delivery of other activities See Fig. 1.

Interview data indicated how individual decisions were made about the feasibility of delivering activities identified as essential in the trial service specifications. If the PIP was not an employee of the GP practice, this impacted on their willingness to undertake some activities e.g. authorising repeat prescriptions, ‘*actually for six months for me being there … it is kind of impossible I think that bit’* PIP19. Similarly, the perceptions of the PIP’s competence, both by the PIP and other stakeholders was a factor whether a new prescription was initiated, ‘*where there might be a clinical call, I think she’ll leave that to the GP to sign*’ GP14.

**Fig. 2:**
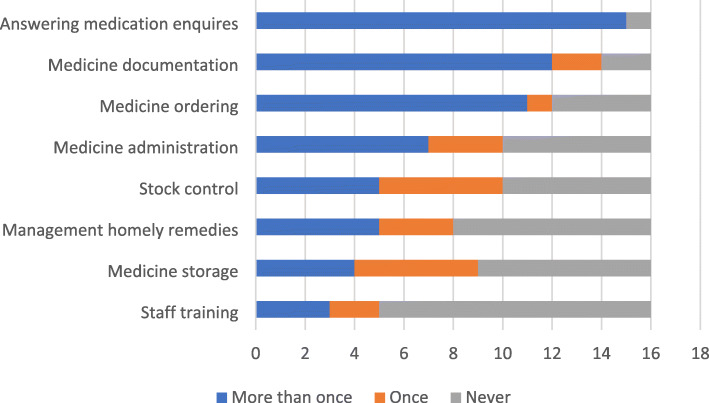
CHIPPS Process evaluation :Topic guide

Information on delivery of ‘discretionary activities’ was only asked in the PIP questionnaires, not those completed by other stakeholders (see Fig. 2). The most frequent activities reported were answering medicine-related enquiries, supporting medicine documentation and medicine ordering.

Interview data and open text questionnaire responses from the three stakeholder groups provided insights into the variation in delivery of discretionary activities. While one pharmacist spent a whole day undertaking stock control after identifying gross over-ordering of appliances, many others described this as an activity that could be carried out by a pharmacy technician. Some care homes reported they did not need some services such as management of homely remedies or staff training, (see Strand 2 focus on mechanisms of impact).

#### Quality of medication review

Review of 105 PCPs, by trial geriatricians, found no overall safety concerns. They explicitly agreed with or made no comment for 49 (46.7%) and 36 (34.3%) PCPs respectively. Seven (6.7%) PCPs were classed as having ‘poor PIP documentation’ and in 13 (12.4%) it was noted that there were ‘potential missed opportunities’, for either a general pharmaceutical care issue or a specific falls risk.

### Strand 2: mechanism of impact (objectives 1,2,3,4)

#### Medication changes

##### Therapeutic area

There were 373 PCPs returned for the 449 residents. Of these, three residents withdrew from the study, so the PCP was not included in the analysis (*n* = 370). There were 76 PCPs which were not available for analysis: 25 residents died before a PCP was completed, 44 residents where the PIP did not deliver the intervention, four PCPs were not completed by two PIPs (reason not given), two residents changed care homes before a PCP was completed, and one PCP was lost to follow-up. Of the 370 PCPs analysed, 44 recorded no medication change. At least one intervention relating to a resident’s prescribed medicine was reported in 326 PCPs (88.1%).

Across the 370 PCPs analysed, there were 566 interventions: 284 (50.2%) were to stop a medicine, 95 (16.8%) to reduce a dose, 49 (8.7%) to change a medicine, 60 (10.6%) to start a medicine, 26 (5%) to increase a dose and 52 (9.2%) to initiate monitoring. The main therapeutic areas associated with interventions were the central nervous system 189 (33.5%), cardiovascular 103 (18.2%), gastro-intestinal 89 (15.8%) and blood and nutrition 69 (12.2%). Therapeutic areas with fewer interventions were the endocrine system 41 (7.1%), respiratory system 24 (4.2%) and skin 23 (4%).

##### Impact on falls and drug burden

A number of medicine changes were related to medicines with a propensity to affect falls (the primary trial outcome) (*n* = 185) or with an associated drug burden (*n* = 189.

Seventy-seven of these interventions involved stopping a medicine, and 46 involved reducing the dose of a medicine with an OR for falls of greater than 1.5. A further 15 medicines with a falls OR of 1.51–2 were started and 16 had a dose increase. There were 179 interventions (31% of interventions) which were associated with a reduction in DBI score [23] and 10 with a DBI increase, see Table 2.

**Table 2:** Medicines interventions with propensity to affect falls and drug burden

**Intervention**	**Falls Odds Ratio.**			
	**1–1.5**	**1.51–2**	**> 2**	**All**
	**No (%) of interventions**			
**Stop**	17 (18%)	77 (82%)	0 (0%)	94 (100%)
**Start**	4 (20%)	15 (75%)	1 (5%)	20 (100%)
**Dose reduction**	8 (15%)	46 (85%)	0 (0%)	54 (100%)
**Dose increase**	1 (5.9%)	16 (94%)	0 (0%)	17 (100%)
	**DBI Score**			
	**0.1–0.5**	**0.51–1**	**1.01–1.5**	**All**
	**No. (%) interventions**			
**Stop**	81 (61.4%)	51 (38.6%)	0 (0%)	132 (100%)
**Start**	3 (50%)	3 (50%)	0 (0%)	6 (100%)
**Dose reduction**	26 (55.3%)	21 (44.7%)	0 (0%)	47 (100%)
**Dose increase**	4 (100%)	0 (0%)	0 (0%)	4 (100%)

Interview data indicated that the majority of GPs were highly supportive of the PIP making medication changes: ‘*Having a pharmacist who understands the process of de-prescribing round the table, is a great asset to our team’,* GP 21. However, two PIPs were hindered in making medication changes due to reluctance of the GP or care home staff to endorse this activity: *‘The GP occasionally re-prescribed things I had stopped- one patient had not had Gaviscon for over 1 year, so I stopped it and the week after the GP had put it back on prescription’ PIP 13.*

The review of PIP prescribing activity against GP records at six-month follow-up indicated that the majority (531, 93.8%) of PIPs introduced changes which remained in place post-intervention. The highest categories of sustained interventions were for medication discontinuation (276 [48.8% interventions) followed by dose reduction (89 [15.7%] interventions).

##### Potential missed opportunities

Of the 185 PCPs reviewed by the research pharmacists, only 27 (14.6%) PCPs were considered to have missed a potential opportunity to make a medication change which could have reduced the resident’s fall risk. These all related to non-review of one or more medicines which had an anticholinergic effect; of the 64 occurrences (27 different medicines), 17.2% had an OR for increased falls risk of 1–1.5, 81.2% of 1.51–2, and 1.6% > 2. A further 38 PCPs (20.5%) were considered to have missed a potential opportunity to optimise the prescribing of other medicines not associated with a falls risk. This was either a need for monitoring such as blood tests, no recorded indication or no recent review. Dates on the reviewed PCPs also indicated that it was several months after the start of the intervention before some residents were reviewed by the PIP. This was confirmed in interviews when PIPs explained it could be a few weeks before they had reviewed all residents and it took time to build a relationship before they could start deprescribing. Where a PCP was reviewed by the geriatrician and the pharmacist researcher, there was strong agreement between comments and potential missed opportunities.

#### PIP support for care home

As part of their role, PIPs could offer general support to the care home including staff training, and supporting good quality communication between care home, GP and community pharmacist.

##### Services provided and frequency

PIP activity logs show that two-thirds of PIP time was spent on resident-related activities. Converting the minutes recorded to approximate hours, this equated to: resident-related face-to-face activity for 348 h (24% of time); resident-related desk-based activity 601 h (43% of time); general tasks for 338 h (24% of time); and travel for 137 h (10% of time). Additional file 5 reports activity type and duration across phases.

In the questionnaire responses, eight of the 16 PIPs reported the time allocation as sufficient, three as too much, and five as not enough. The number of care homes or number of allocated residents for the PIPs did not appear to be associated with overall activity time. Nonetheless where the PIP dealt with more than one care home, they reported challenges in allocating their time between sites: ‘*I had two care homes so I found splitting my time between them, when I didn’t have an even number of participants in each was quite difficult’* PIP2. A couple of PIPs and care home managers reported that towards the end of the intervention, there seemed little for the PIP to do: ‘*I enjoyed that at the beginning, sadly that got a little bit repetitive towards the end because obviously we’d gone over the same residents every single week and there wasn’t any more we could look at ’* CHM 9. However, the majority wanted the intervention to continue.

##### PIP training for care home staff

PIPs were provided with six training PowerPoint presentations to deliver to care home staff. These were not widely used: no PIP used either the sedative or antibiotic training presentation; two PIPs used both the laxatives and pain control training presentation; three PIPs used both the antipsychotics and medicines administration training presentations. In interviews the main explanation for limited use was that the care home already had training in place: *‘both of the homes were with* [name of supplying organisation] *and they do training, a lot of it is online so they’d access to all that, I would have done it if they wanted, but didn’t have the need’* PIP 21*.*

Informal education took place when PIPs worked alongside care staff advising on medication routines.

#### Usefulness of service

In questionnaire responses, when PIPs (*n* = 16) were asked whether they agreed the intervention was worthwhile: 3 ‘strongly agreed’, 4 ‘agreed’, 6 ‘neither agreed nor disagreed’, 2 responded ‘not relevant.’ PIPs were also asked how satisfied they were with the intervention: 5 ‘very satisfied’, 7 ‘satisfied’, 3 neutral’, 1 ‘not at all satisfied’. They provided open text responses in the questionnaire and responded to the interview question on the most and least useful parts of the intervention. There is no data as to why two PIPs responded ‘not relevant’ when asked about worthwhileness of the intervention. Open text and interview comments relating to the usefulness of the service predominantly related to improved medication safety, or improved relationships including contact between PIP and care home staff and residents. Statements about lack of usefulness predominately centred on the complexity of research paperwork. Two PIPs stated ‘A lot of time was spent on a small number of patients’ and one found working across two care homes time intensive.

In questionnaire responses, when GPs (*n* = 8) were asked whether they agreed the intervention was worthwhile: 1 ‘strongly agreed’, 4 ‘agreed’, 2 ‘neither agreed nor disagreed’, 1 ‘strongly disagreed. GPs were also asked how satisfied they were with the intervention: 2‘very satisfied’, 4 ‘satisfied’, 2 ‘neutral’. When the intervention was perceived to be useful it was in factors such as increasing safety of medicines in care homes and reducing wastage. Changes in practice were reported, including in one triad where there had been challenges with implementation: *‘I actually did start to change my practice - when I prescribed a cream for a course of treatment I actually put on the directions ‘discard after a week’ or ‘discard after a fortnight”* GP 19*.* However, the same GP strongly disagreed that the intervention was worthwhile in the questionnaire data stating *‘The care home would not buy into the service and were very angry and dismissive of any interventions and I was the one who had to bear their grumbles’* GP 19*.*

In the two questionnaire responses from care home managers, both agreed the intervention was worthwhile and both were satisfied. During Interviews many care home participants highlighted improved links between care home and the PIP as useful for the staff: *‘it’s actually lifted some stress, because when you’re constantly waiting on calls back and your resident is maybe deteriorating, the residents have really benefitted from it, and that’s what’s important’* CHS 14–*1.*

#### Quality of communication

##### PIP relationship and communication with GPs

In questionnaire responses, PIPs (*n* = 16) rated their relationships with GPs as: 11 ‘very good’, 1 ‘good’ and 4 ‘neutral’. All who rated it neutral were not employed by the GP. Interview data suggested several found it difficult to develop a good relationship within a GP practice previously unknown to them*: ‘level of interaction was quite minimal … the PIP was able to communicate to me via computerised system … I don’t really know the PIP to be honest’* GP 6.

Where the PIP was already employed in the GP practice, there were established working practices and the PIP appeared proactive in reducing GP workload, acting as an autonomous practitioner: *‘the big decisions I told GP about, the little decisions I didn’t bother telling them, it was in the notes anyway’* PIP 9.

##### PIP relationship and communication with care home managers and staff

In questionnaire responses, PIPs (*n* = 16) rated their relationships with care home managers as: 9 ‘very good’, 4 ‘good’, 1 ‘neutral’, 2 ‘difficult’. Where the relationship was difficult, this was associated with poor communication of study purpose: *‘In the care home that was even more apparent that they did not know why I was there for! They said “sorry there is no one you can talk to”*’ PIP 19. Communication was disrupted when the care home manager changed. Eleven care homes had a change of manager at least once during the interventions.

In questionnaire responses, PIPs (*n* = 16) also rated their relationships with care home staff as: 5 ‘very good’, 9 ‘good’, 1 ‘neutral’, 1 ‘very difficult’. Lack of continuity of care staff and challenges in language and understanding affected communication: *‘sometimes I find leaving messages doesn’t always get a result, … that might be a result of a disorganised Nursing Home … communication is difficult, language barriers’* PIP 20*.*

In a small number of triads, the intervention and role of the PIP appeared not to be fully understood (or embraced). In these homes, either the care home appeared reluctant to have any outside agencies coming in, or the care home manager circumvented the PIP to liaise directly with the GP. Several PIPs reported being constrained in pushing forward substantial system changes as many homes had good community pharmacy support and the PIP was aware their time in the home was limited: ‘*I never got to the stage where the (care) home were calling me about things, … it takes at least six months, if not a year, to properly integrate; for all the team members to trust you especially if they have never had a pharmacist working for them … they just don’t know what you do’* PIP 6.

##### PIP communication with residents and residents’ families

Many residents lacked capacity and had communication difficulties. Where PIPs could engage with residents in sharing decision-making about medicine review, they reported this as a largely positive experience. However, the one resident who received regular appointments with secondary care professionals to manage their health condition asserted they did not want the PIP changing their medicines. In interviews, a few care home staff reported that improvements in the residents’ quality of life due to changes in medicine were observable and also appreciated by relatives: ‘*I don’t think I have had any falls from her for a couple of months whereas we were having three or four a week so that is a huge reduction … her husband is much more positive about his experiences when he visits her*’ CHM 9.

##### PIP communication with community pharmacies

In questionnaire responses, PIPs (*n* = 16) rated their relationships with community pharmacists as: 8 ‘very good’, 3 ‘good’, and 5 ‘neutral’. When the PIP was only in-situ for the trial there was an acute awareness of not changing systems already in place: ‘*I was very aware that it was a finite time so I really didn’t want to step on any toes, whereas if you have already built that relationship, they know that you are not coming in to judge them’ PIP 19*. Where the community pharmacist was known to the PIP, communication systems were well-established, and it was seen as a joint endeavour: *‘PIP knows them all [*community pharmacy*] they’re a good team’* GP 14*.*

### Strand 3 outcomes (objectives 2,4)

#### Outcomes of medication review

Medication reviews undertaken in the intervention benefitted care home medicines practice. In interviews, all PIPs reported reducing medicines with anticholinergic and sedative activity, rationalising Medicine Administration Record (MAR) charts, removing no longer-used lotions and ‘as required’ medicines. This activity simplified ordering and dispensing, and reduced risk of medicines being inappropriately used or being beyond their recommended expiry date. Several GPs spoke about the PIP improving safety by having detailed knowledge of residents.

#### Perception of service

Perceived improvement to resident care was the overriding aspect of satisfaction across all stakeholders. Table 3 provides examples of stakeholders’ perception of the CHIPPS service (Table 3).

**Table 3:** Qualitative examples of stakeholder perceptions of the intervention

	PIP	GP	Care homes
**Resident quality of life**	‘It was just pushing it a bit further, looking at the patient as a whole, and being able to do a little bit more and involved the families … made me be more thorough as a prescriber and a pharmacist’ PIP 22 *‘A lady was finding it very hard to swallow medication and I was able to get her medications changed. Some of them were de-prescribed and some of them were changed into a form, a more dispersible form and she was much happier with that’ PIP 1*	‘I don’t have as much time to go round every single one unless it is doing a Care Plan. The Pharmacist looked at them and thought ‘well they have been on this stuff for a long time’ liaised with the staff and said ‘look do you think we might be able to reduce it?’ You know ‘what’s their behavioural, you know their behaviour like?’ and then we have trialled reductions of things and the same with anti-depressants and things as well’ GP16	‘A lady she didn’t particularly like the texture and the taste of the chalky Ad Cal tablets so PIP changed her tablet and that was a good positive experience for her because she was engaged’ CHM 1 ‘We are having good conversations it is not just somebody instructing on us what they think should be done … you are having that dialogue and explaining about your resident and the Pharmacist has taken that into the background of the resident’s care needs and what the difficulties have been … it has been a positive impact’ CHM 12–03
**Increased safety**	‘Some patients were on meds and they hadn’t been necessarily reviewed, so they were then reviewed, so I’d like to think that the quality was better … hopefully the patient care was better’ PIP 2	‘From a safety and medicine waste point of view things have much improved. Care home teams greatly appreciated time taken looking at repeat meds, ensuring they are up to date, have good instructions’ GP 3 ‘It brings another layer of safety to prescribing in a care home, because we all know that that can be a little bit, not unsafe, but it can be challenging, partly because there are so many residents with poly pharmacy’ GP11	‘Just a safety blanket’ CHM 14–02 ‘PIP did ensure that the bloods were taken so that we were getting a true thyroid reading for the dosage. Diabetics as well, we did have urine testing and extra blood tests done on a couple of them’ CHM 6
**Improved medication systems**	‘Stock can go out of date and then it is disposed of and wasted … less of that is happening now’ PIP 8 ‘We have a two-way conversation now. When medicines change, we tell the pharmacy so they know to expect the change’ PIP 9		‘It is meant to be on repeat prescription, you’ve got to go through the rigmarole of phoning the doctor, they’ve got to phone you back, that can take ages, and it’s just a bit of a waste of their time but now the PIP will make sure that it goes on the repeat prescriptions, so there’s never an issue for the next cycle, and things don’t get missed’ CHM 14–02
**Impact on workload**	‘I would have liked to use the training materials for the care home staff, although they were all very experienced and one of the homes was a nursing home with trained nursing staff. I may still do so if the opportunity arises or if they feel it would be beneficial’ PIP 4	‘I want it back! It was very helpful for me; it did take some of the workload off the weekly visits that were all about medication’ GP 16	‘It made ordering easier, a little bit simpler, put the MAR charts into place a bit better, in respect of the things that were on there that were no longer needed they were taken off and things that maybe some of the residents didn’t need’ CHS 19
**Professional development**	‘Really rewarding, as a pharmacy professional and especially as a Pharmacist Independent, I can really use my skills to benefit the Care Home residents directly’ PIP 16 ‘I have the confidence to go in and use the training … I feel confident in their prescriptions that everything has been well looked after, so I would be confident to continue to reauthorize the issue’ PIP 1		‘The care home nurses assessment are also being taken as a valuable tool as well,, and the nurses are liaising with the PIP, to prescribe what they think is needed, I think it’s a win, win situation, the nurses are feeling valued, and the PIP as well’ CHM 21–02 ‘I can’t even remember that far back but I think we already had training, allocated training, delivered by our Community Pharmacist in place anyway so I maybe did turn it down if we were offered it’ CHM 1
**Dissatisfaction with intervention**	‘I think the CHIPPS probably works best if you are actually familiar with the surgery’ PIP 19 ‘I found the pharmaceutical care plan quite cumbersome and I didn’t find it intuitive, I’ve done medication reviews and pharmaceutical plans for quite a few years, and the ones I’ve used a lot more simplistic, Yes it was very comprehensive, there was a lot of information stored on it, for a working document for a pharmacist, it would be alright, but for presenting to a GP it wouldn’t be any use’ PIP 11	‘Occasionally PIP would pick up something that I would have to then address and very often when you try and get blood tests on these patients, they don’t like it and refuse to let the District Nurses near them so it just adds, those little contacts add up and I feel at the end of it I had achieved nothing’ GP 19 ‘It was very useful when there was some communication between the PIP and myself, the negative would be it just didn’t happen enough’ GP 6	‘She was helpful when here but did not attend every week’ CHM 6 ‘I don’t know that it actually happened umm the residents in question that actually agreed to sign up for it their medication was never really appropriate for review so that is perhaps why it never happened’ CHM 18–02

At interview all PIPs reported they enjoyed the opportunity to use their professional skills as an independent prescriber and to have the time to review medications. Many also highlighted the positive experience of having time to be involved with residents and their families. However, lack of care home staff time and staff turnover limited the ability of some PIPs to deliver the service: *‘it was quite difficult because the manager left, and there was a lot of locum staff in from the agency, and it was actually quite difficult at times to speak to staff that knew the patients’* PIP 22*.*

Several GPs mentioned that it had reduced their workload as they could be confident medications were up-to-date and the PIP responded to care home queries. A couple of GPs indicated that similar services were already being routinely implemented: *‘we are going to be employing a pharmacist as part of the Primary Care Network. It is unsure what their role is going to be, but I think certainly they will review the patients who have the most polypharmacy’* GP 8*.*

All care home managers and staff reported they were pleased the PIP medicine review led to reduction of items on the MAR charts which made ordering and dispensing easier, and improved stock control. They appreciated that medication queries were addressed more swiftly, and they had continuity of contact with one practice staff member. Many were able to recall improvements for residents because of more personalised medicines review.

### Strand 4 contextual factors (objective 1, 2, 6)

A contextual factor that impacted on implementation was whether the PIP had previously worked in the GP practice. Where a new relationship had to be developed there were more reports of uncertainty about role and challenges in effective communication such as finding time to meet with the GP and practice staff being unaware of the PIP role: *‘I found it very difficult that I was not an employee of the GP practice. This meant having to start from scratch building relationships with the lead GP for the care home … I was not a known entity in the practice’* PIP 6. There were difficulties getting prompt access to IT systems which meant PIPs were repeatedly transferring data across paper and IT systems. Communication between PIP and GP could be adversely affected by working patterns, especially if both were part-time, but the location and size of the GP practice appeared to have no effect on intervention delivery.

The type and size of care home did not appear to be important in respect of intervention implementation. However, when the PIP dealt with more than one home, they found it difficult to manage their time across sites, as previously noted. Conversely, another PIP, who had close links with the care home before the trial, found it difficult to define any additional activity to undertake during the trial. Care home staff changes disrupted relationships.

**Fig. 3:**
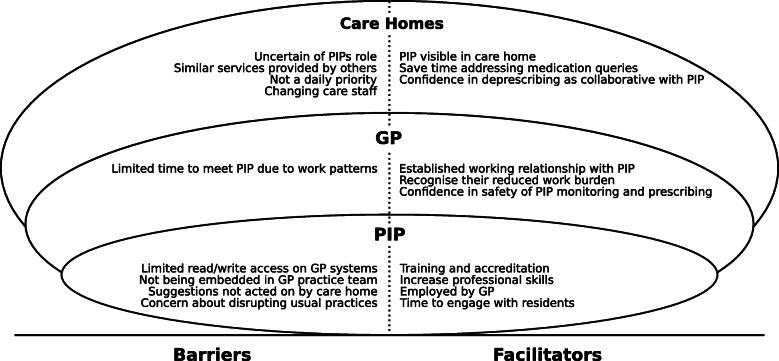
Have a lower case c for coding summary

The facilitators to delivering the intervention centred on trustful relationships and when each stakeholder could see improvement in either systems and/or resident outcomes. The visibility of the PIP in the care home was also an important factor in providing care home staff with a direct access point for medicine requests and queries. Barriers to delivery of the intervention centred on PIP, GP and care home staff ‘s uncertainty about PIP clinical competence. Care home staff had concerns related to impact of reducing drugs on residents’ behaviour. When there was limited communication between stakeholders and a lack of clear demarcation between professional roles PIPs experienced challenges in implementing changes. Figure 3 summarises the barriers and facilitators.

## Discussion

This mixed method process evaluation examined an intervention that placed pharmacist independent prescribers in UK care homes, they took responsibility for a resident’s medicines with a primary aim to mitigate risk of falls in residents and improve their quality of life. A secondary aim was to promote good practice in medicines management. We found that the CHIPPS intervention could be safely implemented; across all data sets it was perceived to improve resident care and safety through direct changes to medicines, and care home staff valued PIPs being able to respond quickly to medicine queries. These staff also reported improvements in medication processes related to ordering and storing.

Variation was seen in the experience of PIPs, number of residents and number of care homes where they delivered the intervention. Nonetheless, stakeholders reported perceived improvements in medication practice and improvements were clearly apparent in some residents’ quality of life. All PIPs, who delivered the intervention, were able to implement most of the essential elements of the service specification and most PIPs implemented some features of the discretionary elements. For those PIPs not already embedded in the GP practice, more barriers were seen, especially as PIPs had a pivotal role in communicating their role in the care home and GP practice. In practices where the PIP was employed their bought-out time supported their additional care home prescribing activity.

We examined the delivery of the intervention in terms of quality, quantity, adaptation and variations across triads. The intervention was consistently delivered, with PIPs providing essential tasks from the service specification to the majority of residents. Variation in delivery was attributed to resident and care home need. Review (by PhD student and research team pharmacists) of residents’ PCPS identified that PIPs had decided no medicine change was required for some residents. Delay in carrying out review of some residents’ medicines left less time for any benefits from a medicine change to be captured within the 6 months’ study period. The review of PCPs by the research team pharmacists indicated that PIPs missed few potential opportunities to change a medicine, possibly reflecting the appropriateness of the bespoke, empirically grounded training [14] in giving PIPs the knowledge and confidence to deliver the intervention.

The PIPs deprescribed a range of medications and reviewed PRN medications. This activity reduced the drug burden for the resident and led to the clearer MARs charts reported by care home staff. Previous studies have also reported increased safety of medicine administration when a pharmacist has undertaken medication review [29, 30].

In relation to mechanisms of impact, PIPs rarely delivered formal training to staff, which was a discretionary activity in the service specification. PIPs had been provided with training material designed to enable care home staff to consider the risk of certain medicines and the distinct needs of older people, but few delivered this training due to care homes reporting lack of need. Yet this might be challenged. For example, the antibiotic training package was never used, yet antibiotic prescribing rates are reportedly high in care homes [31], contributing to the global public health challenge of antibiotic resistance. Similarly, Coon et al. [32] in a review of interventions to reduce inappropriate antipsychotic medications in care homes found staff education effective in reducing prescribing of these drugs. Further work is needed to explore the impact of care home staff training on resident wellbeing, including whether generic training offered by national providers differs in content and outcome from training a pharmacist could tailor for a specific care home.

Several individual components impacted on the perceived effectiveness of the intervention. To enable the PIP fully to deliver the service specifications, other stakeholders, primarily the GP and care home staff, had to understand the PIP role and have confidence in the PIP’s ability. Successful integrated working requires trust between health professionals [33]. We found when GPs had established relationships with the PIP through previous employment, they were more likely to support pharmacist independent prescribing. Some GP practices planned to continue with the expanded pharmacist role post-intervention, suggesting there was acceptance of the intervention. When the PIP had not worked with the GP before, greater negotiation was needed on formalising the scope of PIP prescribing and where this was not achieved accounts of the usefulness of the intervention are more negative from all stakeholders. Our development work suggested each person involved in the care of the resident needed to have a shared understanding of the professional competencies of the PIP and an understanding of their role [12], and that GPs would prefer to work with people who they knew and trusted. Previous studies have reported that where GPs are not familiar with pharmacist prescribing initial acceptance of the role was low [34, 35]. However, increasing demand on GP workloads has seen exponential growth in pharmacists within GP practices, so an extension to their role is likely to be increasingly established [34]. .There will remain a need to avoid overlap in roles which was identified as a concern by a small minority of GPs.

Care home teams were predominantly in favour of the intervention, in particular the ready access to the PIP which facilitated prompt resolution of any prescription queries. Tangible positive outcomes are important when implementing practice change in care homes, particularly where there may be a reluctance to change long-standing medication [36]. Here the CHIPPS intervention was successful as PIPs had dedicated time in the care home each week and care staff had confidence that if changes in medication were not effective, this could be readily discussed with the PIP or reversed. A change in care home managers often led to the leadership in the care home not ‘buying’ into the intervention. There was strong evidence that where care home staff were unsure of the intention of the intervention there was reluctance to fully engage and there were occasional attempts to block pharmacist review. This resonates with an evidence synthesis of care home readiness for healthcare interventions which identified the importance of time to build relationships and the management style of the care home manager as being important for successful intervention implementation [37]. The involvement of a resident’s GP and/or pharmacist is a common factor in successful implementation of interventions in nursing homes [38]. .Further research is needed to examine care home staff’s reluctance to engage with medicines quality improvement activities, as ‘buy in’ from care homes is essential for any clinical intervention.

### Strengths and limitations

A strength of this evaluation is that we collected data from three UK countries with different pharmaceutical and health care systems and therefore, conclusions on the acceptability of the intervention are likely to be widely applicable. The questionnaire and interviews were undertaken after the intervention, but by triangulating these data with study records (PCPs and activity logs) we were not solely relying on participants’ recall. Our results add to the growing evidence that pharmacists can improve care home medication safety through having dedicated time to undertake both review of residents’ medicines and care home medication systems [30, 39].

It is likely that those who were recruited to the trial could be early adopters of new practice and as such, more receptive to change and innovation. It is noted that those who volunteer for interview may have stronger positive or negative experiences than those who do not. A review of patients’ opinions when pharmacist consultations were initially instigated in GP practices found patients had limited understanding of the pharmacist’s role and often had stronger preference for their own GP service, but we were only able to interview one resident, in part due to COVID-19 restrictions at the end of the study. Challenges in collecting data from these stakeholders also lay in the cognitive ability of residents to consent to interview and relying on care home staff to be gatekeepers to residents and their family. The views of residents and families need to be further examined as there may be differing expectations on who has the clinical expertise to prescribe.

### Practice implications

We found that it was acceptable to have a prescribing pharmacist as a named person with responsibility for care home resident medicines management; a person who would also provide a link between care home and GP. Most stakeholders reported positive outcomes related to resident well-being and improvements in medicines management. However, there are areas which could be developed to support implementation of this role.

The service was set up with each PIP providing one weekly session to each home. Experience showed that after residents had been reviewed, care plans had been implemented, and general medicine management processes streamlined, the amount of time required to deliver the service diminished. However, as one of the valued outcomes for the care homes was knowing and being able to depend on the pharmacist, visits should be sufficiently regular to maintain that relationship, answer emerging queries and review the medicines of any new residents.

As primary care pharmacy provision transitions with the introduction of Primary Care Networks [40] there needs to be a more comprehensive consideration of which health and social care professionals might be best placed to undertake medicines management with care home residents. Our study has illustrated that prescribing pharmacists have the skills to fulfil this role, and GPs and care home staff are likely to find this acceptable. PIPs liaised with community pharmacists to discuss medicines management, MAR chart errors and to keep them informed of medicine changes, thereby ensuring efficient medicine ordering. While some PIPs undertook store cupboard stock control, this activity might be more cost-effective for a pharmacist technician to carry out. The roles of community pharmacists and pharmacy technicians need to be considered when planning pharmacist-led care home medication management.

Interlinked IT systems would streamline clinical reporting and reduce duplication. PIPs often worked on paper records in care homes, having to manually transfer all activity into electronic GP databases. Furthermore, interlinked IT systems would enable other members of the primary care team, such as community nurses and paramedics, to access up-to date resident medicine information. Such improved communication across the multidisciplinary team could potentially enhance future quality of care for residents.

## Conclusion

This process evaluation has found that the CHIPPS intervention, giving a pharmacist independent prescriber responsibility for management of care home residents’ medicines, can be successfully implemented as intended. The evaluation found no serious adverse effects when pharmacists led on prescribing and deprescribing and indeed, care home staff often perceived an improved quality of life in residents, and most were fully supportive of the intervention. There was widespread deprescribing across therapeutic areas, with a number of medicine changes related to medicines with a propensity to affect falls. Generally, GPs and care home staff had confidence in the pharmacist’s competence and there was general agreement that this was a useful intervention, which increased medicine safety Where the PIP had not worked previously with the GP, they needed to actively build the relationship as without this the PIP experienced barriers and resistance to their role in GP practices and care homes.

## Supplementary Information

**Additional file 1.** CHIPPS Intervention Service Specification

**Additional file 2.** CHIPPS Process evlauation: Topic guide.

**Additional file 3.** CHIPPS study coding summary.

**Additional file 4.** Demographics of triads and participants in the CHIPPS study.

**Additional file 5 **PIP Activity use across phases *-* nature and duration in minutes.

## Data Availability

The datasets used and/or analysed during the current study are available from the corresponding author on reasonable request.

## Abbreviations

CHM: Care Home Manager
CHS: Care Home Staff
DBI: Drug Burden Index
GP: General Practitioner
NoMAD: The Normalisation MeAsure Development questionnaire
OR: Odds ratio
PCP: Pharmaceutical Care Plan
PIP: Pharmacist Independent Prescriber
