# Supplementary material for: Process evaluation for the Care Homes Independent Pharmacist Prescriber Study (CHIPPS)
Source: BMC Health Serv Res. 2021 Oct 2;21:1041. doi: 10.1186/s12913-021-07062-3 (PMC8487235; doi:10.1186/s12913-021-07062-3)
Supplement: Supplementary file 1 — Additional file 1. CHIPPS Intervention Service Specification [file 12913_2021_7062_MOESM1_ESM.pdf]

Additional file 1 CHIPPS Intervention Service Specification

	Specification
**Recruitment and employment of the Pharmacist Independent Prescriber (PIP)**	
	Excellent interpersonal, communication and IT skills
	Familiarity with relevant GP software systems
	Experience of providing prescribing and medicines management advice and support
	Previous experience of working in GP practice environment
	Be able to travel to site locations
	A mobile phone to be contactable for the purposes of delivering this service
	Appropriate indemnity insurance for prescribing
**PIP roles and responsibilities**	
Review each resident's medication and develop and implement a pharmaceutical care plan (essential)	Optimise prescribing ensuring clear indication and evidence base for each medication (taking into consideration national and local pathways, guidelines and formularies), informed by tools such as STOPP/START
	Minimise the potential for adverse effects
	Optimise the dose of all medication
	Co-ordinate appropriate monitoring and associated tests for all medicines and conditions

	Agree initial care plan with GP, care staff and resident (where appropriate)
	Document and maintain records relating to review and care plan in GP and care home records as appropriate
Prescribing (essential)	
	Authorise repeat prescriptions
	Co-ordinate appropriate monitoring and associated tests for all medicines and conditions
	Deprescribe medicines according to agreed pharmaceutical care plan
	Document medication changes in GP and care home records and notify supplying pharmacy of all changes to medication within 24 hours
	Only initiate new medicines for existing diagnoses or for common ailments which can be managed with medicines classified by the Medicines and Healthcare products Regulatory Agency (MHRA) as Pharmacy (P) or General Sales List (GSL)
	Any additional areas of prescribing must be agreed and documented with the GP practice prior to prescribing (e.g. antibiotics for simple UTIs
Communication (essential)	
	Agree local protocols for communication with GP practice and care home prior to commencing service. This should include:<br>○ Process of communication and messaging

	○ The location and expected level of detail of all PIP interventions in the medical records Process and communication of referrals for activities outside the competence of the PIP
	Inform supplying community pharmacy about service and role (prior start of service)<br>○ Communicate all changes in medication to supplying pharmacy
	Complete all documentation and recording of activities as required by the study team.
Support systematic ordering, prescribing, and administration processes with each care home, GP practice and supplying pharmacy where needed: (undertaken at PIP's discretion)	
	Provide instructions on how to administer each drug
	Synchronise residents prescription quantities for monthly cycles
	Add or clarify directions for all medication where it is currently not clear
	Provide advice on repeat prescription ordering processes to:<br>○ Minimising missed items<br>○ Optimising quantities
	Optimise the use of homely remedies within the care home
	Reconcile resident medication following a transfer of care
Training provision (undertaken at PIP's discretion)	
	Review training needs of care home and GP practice and draft proposed training plan

	Provide training to care home staff on training needs basis from agreed list of potential topics/areas
	Provide guidance to relevant GP practice on training needs basis from agreed list of potential topics/areas
**Safe and effective service provision**	
	PIP will be contactable and respond to messages within 24 hours (Monday - Friday)
	The PIP will establish a locally agreed protocol with the GP practice for referral/notification of all medicine related queries from CHIPPS participants to the PIP as appropriate (see 4.3.5)
	PIP will have full (read/write) access to GP record system to issues prescriptions and update records
	Where possible PIP will use remote access to update records when changes are made to GP held record<br>o Where remote access is not feasible the PIP must update records within 24 hours of making a change
	PIP will have full (read/write) access to care home records to update records during all visits using appropriate local reporting systems
	The PIP will visit/contact the care home at least once a week
	The PIP will visit/contact the GP practice at least once a week
	Wherever possible, all annual leave should be agreed before the beginning of the study. A clear system for transfer of responsibility communicated to GP, care home and supplying pharmacy

	The PIP will work within the local prescribing formularies of GP practice and primary care organisation.
	The PIP will report and document all significant clinical events or near misses using local reporting procedures and study documentation.
	Ensure all records are aligned
