# Supplementary material for: Process evaluation for the Care Homes Independent Pharmacist Prescriber Study (CHIPPS)
Source: BMC Health Serv Res. 2021 Oct 2;21:1041. doi: 10.1186/s12913-021-07062-3 (PMC8487235; doi:10.1186/s12913-021-07062-3)
Supplement: Supplementary file 3 — Additional file 3. CHIPPS study coding summary. [file 12913_2021_7062_MOESM3_ESM.pdf]

CHIPPS study Coding summary

| Main code | Sub code | | Number of participants in that code | Number of times a code mentioned by participants |
|---|---|---|---|---|
| **Implementation** | | | 25 | 166 |
| | Care home study procedure | | 18 | 61 |
| | GP study procedure | | 11 | 26 |
| | Services provided by PIP | | 14 | 43 |
| **Mechanisms of Impact** | | | 19 | 114 |
| | Biochemical monitoring | | 14 | 17 |
| | Care home workload due to interventions | | 11 | 38 |
| | Medication changes made | | 28 | 80 |
| | GP tasking items to PIPs | | 10 | 22 |
| | Staff training | | 20 | 27 |
| | Non patient facing activities undertaken by PIPs | | 17 | 60 |
| | | Authorising repeat prescription | 16 | 24 |
| | | Completing PCP | 9 | 23 |
| | | Tasking items to GPs | 6 | 6 |
| | | Liaising with Community Pharmacists | 13 | 22 |
| | | Liaising with Primary Care practice staff | 7 | 9 |
| | | Review of medication systems | 7 | 8 |
| | | Reviews of stock | 6 | 6 |
| **Outcomes** | | | 24 | 125 |
| | Adding to multidisciplinary practice | | 30 | 81 |
| | Case studies improvement to residents | | 29 | 71 |
| | Case studies planned change not successful | | 10 | 10 |
| | Ordering system changes | | 14 | 27 |
| | Care home satisfaction with intervention | | 15 | 70 |
| | | Mixed | 2 | 2 |

| | | | | |
|---|---|---|---|---|
| | | Negative | 1 | 1 |
| | | Neutral | 1 | 4 |
| 4 | | Positive | 15 | 71 |
| | GP satisfaction with intervention | | 8 | 45 |
| | | Mixed | 4 | 5 |
| | | Negative | 4 | 8 |
| | | Neutral | 0 | 0 |
| | | Positive | 9 | 38 |
| | PIP Satisfaction with intervention | | 14 | 41 |
| | | Mixed | 8 | 8 |
| | | Negative | 4 | 7 |
| | | Neutral | 1 | 11 |
| | | Positive | 11 | 26 |
| **Intervention safety** | | | | |
| | Care home perspective | | 9 | 16 |
| | GP perspective | | 9 | 28 |
| | PIP perspective | | 9 | 21 |
| **Contextual factors** | | | | |
| | Barriers to delivery | | 31 | 86 |
| | Facilitators to delivery | | 22 | 75 |
| | Care home staff awareness of intervention | | 20 | 46 |
| | GP workload impact | | 19 | 47 |
| | Impact of patient factors on delivery | | 16 | 25 |
| | Impact of site factors on delivery | | 25 | 55 |
| **Communication** | | | 25 | 154 |
| | Care home to PIP | | 24 | 96 |
| | GP to CH | | 9 | 11 |
| | PIP to CH | | 24 | 113 |
| | PIP to GP | | 21 | 52 |
| | PIP to others | | 23 | 63 |
| **Intervention normalised** | | | 26 | 59 |

| | | | | |
|---|---|---|---|---|
| | Actions taken by PIPs to ensure normalises | | 10 | 17 |
| | | Adapted working with CH | 18 | 29 |
| | | Adapted working with GPs | 8 | 10 |
| | | Adaptive working with others | 5 | 7 |
| **Professionals roles** | | | 24 | 78 |
| **Legacy of Intervention** | | | 23 | 47 |
| **Geographical difference** | | | 14 | 23 |
