# Supplementary material for: Process evaluation for the Care Homes Independent Pharmacist Prescriber Study (CHIPPS)
Source: BMC Health Serv Res. 2021 Oct 2;21:1041. doi: 10.1186/s12913-021-07062-3 (PMC8487235; doi:10.1186/s12913-021-07062-3)
Supplement: Supplementary file 4 — Additional file 4. Demographics of triads and participants in the CHIPPS study. [file 12913_2021_7062_MOESM4_ESM.pdf]

Additional file 4 Demographics of triads and participants in the CHIPPS study

Triad ID	yr pharmacist	~yr Ind prescriber	CH experience	No residents	Activity~ hrs	size practice < or ≥10K	location	IMD	GP employs PIP	CH type	funded	IMD	No residents	changed managers	CH type	funded	IMD	No residents	changed managers	CH type	funded	IMD	No residents	changed managers	Data collection						
	PIP					GP				Care home 1					Care home 2					Care home 3					Interviews				Questionnaire		
																									PIP	GP	CHM	CHS	PIP	GP	CHM
1	12	1month	N	6	50	<	urban	3	Y	dual	pri	10	33	0											*		*		*	*	
2	21	2months	Y	20	86	≥	semirural	8	Y	dual	pri	8	60	0	dual	pri	8	38	0						*				*		
3	12	1	y	16	69	<	Urban	6	N	res	LA	7	29	0	res	LA	6	35	0							*					
4	20	1	N	24	12	≥	urban	10	N	dual	pri	10	60	0	res	pri	10	34	0						*				*		
5	9	1 1/2	Y	20	88	<	urban	1	N	res	LA	3	89	1																	
6	10	1 1/3	N	22	48	≥	urban	2	N	res	Vol	5	44	0											*	*	*	*	*	*	*
7	23	2	N	9	44	<	rural	8	Y	dual	pri	8	45	0																*	
8	24	1 1/2	Y	24	70	≥	semirural	4	Y	dual	pri	4	29	0	res	pri	8	34	0						*	*		*	*		
9	25	1 1/2	N	21	92	≥	rural	3	Y	res	pri	6	62	0											*		*		*		
10	10	2	N	23	84	≥	urban	4	Y	dual	pri	7	60	0																	
11	30	3	N	14	78	<	semirural	10	Y	res	pri	8	33	0											*	*		*	*		
12	25	4	y	19	68	≥	Urban	10	y	res	Vol	5	84	0	res	pri	10	44	0	res	Vol	10	46	0			*				
13	8	4	N	20	145	≥	urban	4	N	dual	pri	2	83	1															*	*	
14	12	4	N	20	34	<	rural	9	Y	dual	pri	4	33	0	dual	pri	9	27	1	dual	pri	9	18	1	*	*	* *	*	*		
15	10	6	99	18	42	<	urban	1	Y	dual	pri	7	65	3											*				*	*	
16	11	6	Y	20	107	<	urban	1	N	dual	pri	1	76	1											*	*				*	
17	16	7	Y	6	20	missing	urban	3	Y	dual	pri	7	66	0	dual	pri	10	58	0	dual	pri	10	29	0	*				*		
18	31	8	N	9	15	<	urban	4	Y	dual	pri	6	41	0	dual	pri	8	57	1								*		*		
19	32	9	Y	24	99	<	rural	6	N	res	pri	6	47	0											*	*		*	*	*	
20	30	10	Y	23	60	missing	semirural	5	Y	dual	pri	7	48	0	dual	pri	8	64	0	dual	pri	5	35	0	*				*		
21	40	14	Y	21	45	<	urban	10	y	dual	pri	10	35	0	dual	pri	6	41	0							*	* *	*	*		*
22	36	16	Y	11	50	<	urban	10	Y	dual	pri	5	53	1											*				*		
W/D 23	18	1	N	11	missing	<	urban	3	Y	dual	pri	2	40	1																	
W/D 24	8	2	N	24	missing	≥	urban	6	N	res	pri	3	28	0	res	pri	5	24	0											*	
W/D 25	5	1 1/2	N	24	missing	≥	urban	3	N	res	pri	1	98	0																	

Key
CH Care home
IMD Indices of multiple deprivation
* Partcipated in
