# Supplementary material for: Process evaluation for the Care Homes Independent Pharmacist Prescriber Study (CHIPPS)
Source: BMC Health Serv Res. 2021 Oct 2;21:1041. doi: 10.1186/s12913-021-07062-3 (PMC8487235; doi:10.1186/s12913-021-07062-3)
Supplement: Supplementary file 5 — Additional file 5 PIP Activity use across phases - nature and duration in minutes. [file 12913_2021_7062_MOESM5_ESM.pdf]

Additional file 5 PIP Activity use across phases - nature and duration in minutes

	Resident related - face-to-face (no. minutes)	Resident related - desk based (no. minutes)	General Tasks (no. minutes)	Travel (no. minutes)	Total (average minutes per resident)
Pilot (PIP =4)	3903	9567	2616	2960	276
Phase 1 (PIP =6)	7259	9087	5660	1655	189
Phase 2 (PIP =6)	5490	7579	8690	2650	237
Phase 3 (PIP =6)	4255	9830	3305	950	189
Total Minutes	20907	36063	20271	8215	217
**Total (to nearest hour) (%)**	**348 (24%)**	**601 (43%)**	**338 (24%)**	**137 (10%)**	1424 3.6 hrs per resident
